# Longitudinal datasets of health app reviews for privacy and trust modeling

**DOI:** 10.1016/j.dib.2026.112740

**Published:** 2026-04-02

**Authors:** Timoteo Kelly, Abdulkadir Korkmaz, Samuel Mallet, Connor Souders, Sadra Aliakbarpour, Praveen Rao

**Affiliations:** a Institute for Data Science & Informatics, University of Missouri, Columbia, MO, USA; b Dept. Electrical Engineering & Computer Science, University of Missouri, Columbia, MO, USA; c Dept. of Computer Science, Yale University, New Haven, CT, USA

**Keywords:** Digital health, mHealth apps, Text classification, Natural language processing, Cognitive security, Trust, Patient portal, Telehealth

## Abstract

We present Health App Reviews for Privacy & Trust (HARPT), a large-scale annotated corpus of user reviews from patient portal and telehealth applications (apps) aimed at advancing research in user privacy and trust. The dataset comprises 480,450 user reviews labeled across seven classes that capture critical aspects of trust in applications, trust in providers, and privacy concerns.

Our multistage strategy integrated keyword-based filtering, iterative manual labeling with review, targeted data augmentation, and weak supervision using transformer-based classifiers. In parallel, we manually annotated a curated subset of 7000 reviews to support the development and evaluation of machine learning models. We benchmarked a broad range of models, providing a baseline for future work. HARPT is released under an open resource license to support reproducible research in usable privacy, trust and health informatics.

Specifications Table

**Table:** 

Subject	Health Sciences, Medical Sciences & Pharmacology
Specific subject area	Health Informatics / Machine Learning / Social Science / Natural Language Processing
Type of data	.csv (annotated training data), .tab (tabular review data), .md (dataset documentation)
Data collection	User reviews were initially scraped from Google Play Store. A keyword filter isolated privacy and trust concept. 4000 reviews were manually labelled; back-translation (French, Spanish, German) was used to balance the set to 7000 ground-truth reviews. Our model was trained using ground truth and used to label 480k reviews
Data source location	Columbia, MO, USA (University of Missouri).
Data accessibility	Repository name 1: Harvard Dataverse Data identification number: doi:10.7910/DVN/U6OF6F Direct URL to data: https://dataverse.harvard.edu/dataset.xhtml?persistentId=doi:10.7910/DVN/U6OF6F Repository name 2: Huggingface For software and supplemental materials Data identification number: 10.57967/hf/5820 Direct URL to data: https://huggingface.co/tk648/XLNET-base-finetuned-HARPT?doi=10.57967/hf/5820
Related research article	T.Kelly, & J.R. Bautista. Unintentional Information Blocking: A Longitudinal Analysis of Availability Failures in mHealth (2011-2025). In Proceedings at the 14th IEEE International Conference on Healthcare Informatics, DOI:10.5281/zenodo.19366301, 2026.

## 1 Value of the Data

• The HARPT corpus provides a high-quality, 7000-sample gold standard ground-truth dataset for training and testing supervised learning architectures, paired with a massive 480,000-sample corpus that enables large-scale natural language processing (NLP) tasks and longitudinal study of user feedback.
• Every review in the ground-truth subset was subjected to a rigorous three-pass manual annotation protocol (Fleiss’ Kappa = 0.877), ensuring that complex categories such as *Risk, Ethicality* and *Data Control* are represented with high precision (F1 Macro = 0.96) for sensitive health informatics research.
• Spanning 14 years (2011–2025), this dataset allows researchers to systematically track the evolution of patient concerns regarding data governance, provider professionalism, and application reliability during the critical global shift toward telehealth and digital patient portals.
• By providing statistically calibrated confidence scores for every label in the 480k corpus (verified by a 0.001 confidence-accuracy gap), the data empowers users to perform high-certainty filtering, reducing the impact of noise traditionally found in weakly supervised or automatically labelled datasets.
• Because it reflects the real-world prevalence of user concerns where *Competence* and *Reliability* dominate while *Risk* and *Ethicality* are rare, the HARPT corpus serves as a valuable resource for developing and evaluating machine learning methods designed to handle highly skewed data.

## 2 Background

The use of digital health has grown in recent years, with mobile health applications leading with near ubiquitous adoption [1]. Telehealth and patient portal platforms which fall under the umbrella term electronic health (mHealth) are one of the largest areas of growth and change for patient-provider care [2]. Now that telehealth and patient portals have been integrated into mobile devices via applications (apps), patients should have an easier time accessing care. User reviews from these mHealth platforms remain a rich source of unsolicited user feedback to gain insights in patient perceptions [3,4]. This lack of large-scale, annotated datasets specific to privacy and trust has limited the ability of researchers to systematically analyze these concerns using natural language processing (NLP) techniques.

Current work relies on surveys, interviews or datasets that are not publicly available which limits the reproducibility and the ability to track concerns over time [[5], [6], [7]]. We developed the HARPT corpus to address this critical gap, by providing a systematically labeled resource that enables the use of machine learning models to uncover privacy and trust dynamics inherent in over a decade of patient discourse.

The data in this study is classified as secondary data as it consists of pre-existing, user-generated feedback originally submitted to apps on the Google Play Store rather than being actively elicited by the researchers. However, while the raw data originate from a secondary source, our systematic extraction, keyword-based filtering and rigorous manual annotation process transformed this raw public data into a specialized, novel corpus, HARPT.

While previous literature has extensively explored mobile application feedback [3,4], existing app review datasets present significant limitations when applied to the digital health domain [8]. Traditional app review datasets typically classify user feedback into high-level categories [3]. The critical limitation of these datasets is their lack of domain specificity and theoretical depth. They treat privacy as a monolithic issue, if it is captured at all, and entirely fail to account for the nuanced dynamics of Trust in Provider or high stakes nature of protected health information (PHI).

On the other hand, while dedicated trust and privacy datasets do exist, they are primarily derived from structured surveys, localized usability studies or broad social media [9,5,7]. The limitation of these privacy focused datasets is their lack of ecological validity and NLP readiness at scale. Survey data does not reflect the colloquial, unstructured real life language users use in the real world, and manual qualitative coding studies are typically restricted to sample size of a few hundred reviews [10], limiting their utility for training modern large language models (LLMs).

HARPT is the first longitudinal corpus to explicitly operationalize established trust and privacy frameworks into a multidimensional seven-class taxonomy specifically engineered for the unique technical and ethical friction points of the mHealth domain.

## 3 Data Description

### 3.1 Classes description

The HARPT corpus consists of seven classes (see Table 1) of patient trust and privacy concerns identified in mHealth application reviews. The categories encompass three primary dimensions: Trust in Application (TA), Trust in Provider (TP), and Privacy Concerns (PC). Each class represents a specific element of the patient experience, ranging from technical functionality to ethical data stewardship. *TA* is defined as the user’s belief that the application possesses the attributes necessary to perform reliably; *TP*, defined as the belief that healthcare professionals possess the competence and ethical responsibility to act in the patient’s best interest; and *PC*, reflecting user apprehensions regarding the control, protection, and accuracy of their personal health information. Further description for each of these classes as well as examples are provided below.

**Table 1:** The seven class taxonomy framework.

Privacy Concerns	Trust in Providers	Trust in Applications
Data Quality	Ethicality	Reliability
Data Control	Competence	Support
		Risk

**Competence:** Perceptions of provider professionalism and care quality. (E.g, “The doctor I spoke with on this app was incredibly knowledgeable and took the time to thoroughly explain my test results”.)

**Reliability:** User perceptions of app crashes, login failures or inconsistent functionality. (E.g, “I can’t login! Every time I enter my username and password it just loads and loads.”)

**Support:** User experience with help services, communication or materials. (E.g, “Customer support responded to my query within a few hours and helped me resolve my issue.”)

**Risk:** Worries about security, breaches or misuse of data. (E.g, “I’m worried about how the app stores my personal health records.”)

**Ethicality:** Judgments of provider fairness, transparency or responsibility. (E.g, “I love that the app asks for my consent before collecting my data.”)

**Data Quality:** Issues with incorrect, outdated or incomplete health data. (E.g, “The app is showing someone else’s health record!”)

**Data Control:** Concerns about consent, third-party access, control over personal data. (E.g, “It’s great the app lets me review and delete my medical records whenever I want.”)

Our classes are grounded in well-established theories across organizational trust, technology acceptance and privacy psychology. We leverage these constructs into a workable taxonomy, adapted from established theoretical components to fit the natural language expressions in app reviews.

For *PC,* we reference IUIPC [9], ubiquitous computing concerns [11], and healthcare-specific trust models [12,5,8], which motivate the *data control* and *data quality* categories. For TP*,* we build on Mayer et al. [13], who identify ability, benevolence, and integrity as key antecedents of trust, and Hosmer et al. [14], who emphasizes fairness and ethical responsibility. These perspectives map directly to our labels: *competence* and *ethicality*. For *TA*, we draw on McKnight et al.’s work on trust in technology [15], which emphasizes beliefs about a system’s reliability, helpfulness, and functionality, and Gefen et al.’s integration of trust with technology acceptance constructs [16], which highlight how risk, perceived usefulness, and trust in the technology influences user behavior. These theories inform our labels of *reliability, support*, and *risk*.

This framework is uniquely representative of the healthcare app domain because it unifies dispersed theoretical models into a single, cohesive labeling schema tailored specifically for NLP. Prior literature has mostly confined these constructs to theoretical models or surveys. These distinct dimensions capture organically how users communicate support failures, perceived risks, data control issues, and we have successfully utilize traditional privacy and trust psychology for scalable analysis of real world user data.

### 3.2 Data records and file structure

The HARPT dataset is hosted on the Harvard Dataverse repository and is provided in standard, machine-readable formats to ensure cross-platform compatibility. The repository contains two primary data files: the gold-standard manually annotated subset provided in comma-separated values format (ground_truth.csv,), and the large-scale weakly supervised corpus provided in tab-separated values format (HARPT.tab,). Both files utilize standard UTF-8 encoding. Because the data collection and processing phases differed for these sets, their tabular structures vary.

1. Ground Truth Dataset (ground_truth.csv) This file contains the manually annotated reviews and consists of 6 variables:
  • **app_name:** The public name of the mHealth application (e.g., MyChart).
  • **at:** The timestamp of the review submission.
  • **content:** The preprocessed, unstructured text of the user's review.
  • **score:** The user-provided quantitative star rating (1 to 5).
  • **trust_dimension:** The manually assigned privacy or trust taxonomy label.
  • **word_count:** The calculated word count of the review content.
2. Large-Scale Dataset (HARPT.tab) This file contains the weakly supervised reviews and includes expanded metadata, consisting of 16 variables:
  • **reviewId:** A unique alphanumeric identifier for each review.
  • **content:** The unstructured text of the user's review.
  • **score:** The user-provided quantitative star rating (1 to 5).
  • **thumbsUpCount:** The number of upvotes the review received from other users.
  • **reviewCreatedVersion:** The version of the application the user was reviewing.
  • **at:** The timestamp of the review submission.
  • **replyContent:** The text of the developer's response to the review (if applicable).
  • **repliedAt:** The timestamp of the developer's response.
  • **app_name:** The public name of the mHealth application.
  • **app_id:** The unique application bundle identifier on the Google Play Store.
  • **word_count:** The calculated word count of the review content.
  • **year:** The extracted year the review was posted.
  • **App_Type:** The platform categorization (e.g., Patient Portal, Telehealth).
  • **trust_dimension:** The model-assigned privacy or trust taxonomy label (e.g., 'reliability', 'competence').
  • **confidence_score:** The softmax probability score generated by the XLNet model, allowing researchers to filter predictions by mathematical certainty.

### 3.3 Dataset statistics

The HARPT corpus (see Table 2) comprises 480,450 weakly labeled reviews collected from 67 distinct mobile health applications over a 14-year period (2011–2025). The dataset provides comprehensive coverage of the mHealth landscape, with 64.8% of reviews originating from patient portals and 35.2% from telehealth platforms. Five major applications account for over 60% of the total corpus volume: *MyChart* (19.4%), *FollowMyHealth* (14.7%), *healow* (9.8%), *Practo* (9.1%), and *Doctor on Demand* (7.3%).

**Table 2:** HARPT dataset descriptive statistics.

Metric	Value
Total Weakly Labeled Reviews	480,450
Temporal Coverage	2011–2025 (14 years)
Distinct Mobile Health Apps	67
**Platform Review Distribution**	
Patient Portals	64.8%
Telehealth Platforms	35.2%
**Review Length**	
Median / Mean	10 / 16.5 words
Range	1–675

Textual analysis shows that the reviews are generally concise, with a median length of 10 words and a mean of 16.5 words (range: 1–675). The associated user star ratings exhibit a significant skew toward positive sentiment, with 65.4% of users providing five-star ratings compared to 16.7% providing one-star ratings. *Competence* and *Reliability* make up the majority at 44.3% and 27.5%, respectively. *Risk* and *Ethicality* make up the two lowest classes at 1.4% and 1.3%.

## 4 Experimental Design, Materials and Methods

**Fig. 1:**
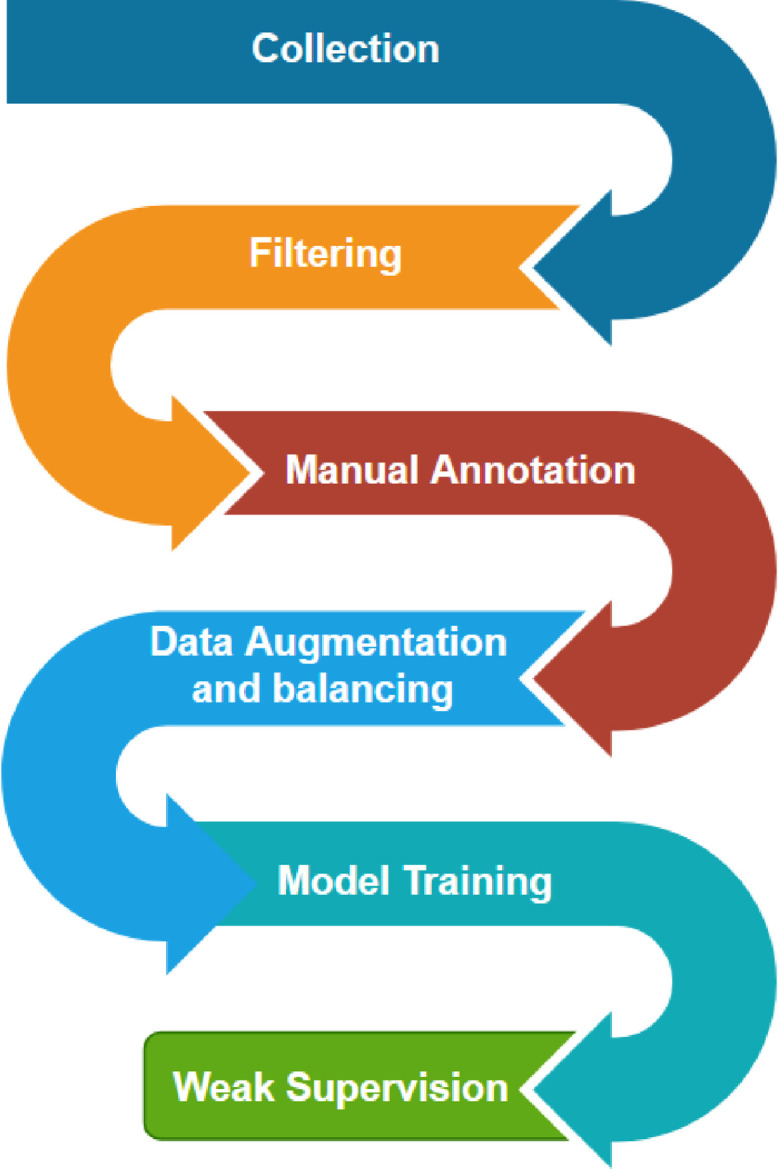
Methodology pipeline showing each step.

The HARPT corpus was constructed through a multi-phase experimental design (See Fig. 1), moving from raw data collection to manual annotation, data augmentation, and finally, large-scale weak supervision.

### 4.1 Data collection and filtering

A total of 457,165 user reviews were scraped from the Google Play Store leveraging the Python packages google_play_scraper and sqlite3 with a custom script in August 2024, covering 32 patient portal applications and 27 telehealth applications. Queries were configured with the parameters, lang=`en’, country=`us’, and ‘Sort.NEWEST’ to systematically capture recent user feedback in English from the United States.

To ensure the dataset was representative of privacy and trust we utilized a keyword-based filtering strategy. We created a dictionary of over 300 domain specific terms mapped to our seven classes (see Supplemental Materials for full keyword list). Matching reviews were extracted and consolidated into a single dataset. We then filtered for reviews that have a word count >8 and <45. This range was empirically selected to exclude uninformative brevity and excessive outliers. From this, we generated a random sample of 4000 reviews.

One of the challenges encountered during this process was the dynamic loading constraints of the Google Play Store, which restricts the number of reviews to a maximum of 200 results per page. We engineered a custom pagination protocol into our scraping script. This was designed to iteratively request, extract and append the 200 review batches, with a randomized 1–5 s sleep interval between iterations to ensure stable data retrieval.

Another challenge was during the processing phase, the highly unstructured, noisy nature of the raw user feedback frequently contained non-standard characters, emojis, duplicates, spam and uninformative fragments. We used pandas and NLTK Python package to mitigate this by systematically removing emojis, duplicate records and normalizing the text data. We further mitigated this by enforcing strict 8–45 word count boundary which filtered out extreme outliers and meaningless brevity.

### 4.2 Annotation protocol and inter-rater reliability

We established a three-pass workflow with four annotators, the 4000 reviews were divided into batches and assigned in a rotating fashion so that every review was independently labeled exactly three times by three different annotators. After one reviewer labeled one set, they were moved to a new set that had been previously labeled by another reviewer until the comprehensive three-pass coverage was achieved. Labels were adjudicated via a majority vote. In instances of three-way disagreement (noted in only one case), the lead researcher acted as the final tiebreaker. We distributed this work over a five-month period (November 2024 to March 2025) to mitigate the risk of annotator fatigue by pacing the annotation process and enforcing a 45-word count maximum per review. This ensured that cognitive load would remain manageable, enabling quality and consistent labeling. This approach yielded a strong inter-rater reliability, achieving a Fleiss’ Kappa score of 0.877, signaling near perfect agreement across the corpus.

The final taxonomy categorized reviews into three meta-dimensions: 1) Trust in Application: Reliability, Support, and Risk. 2) Trust in Provider: Competence and Ethicality. 3) Privacy Concerns: Data Quality and Data Control.

### 4.3 Data augmentation via back translation

We found that reviews relating to Privacy Concerns such as Data Control and Data Quality, and Ethicality from Trust in Provider represented a minority of the overall dataset. To prevent our fine-tuned model from developing majority class bias, we used a synthetic data generation method via back-translation to artificially up-sample the minority classes. This significant class imbalance, showed the Competence category acting as the majority class (n = 1561). Back Translation involves translating the original English reviews into a target language and then back into English to create a similar paraphrase. We translated our reviews into French, Spanish and German. All minority classes were augmented using back-translation. Our majority category, Competence, was under sampled from 1561 to 1000. The BLEU score was calculated to verify the semantic fidelity of the augmented samples, which was an average score of 30.43. This confirms some variation while still preserving meaning.

### 4.4 Machine learning experiments and model selection

The final balanced dataset of 7000 reviews served as the training and evaluation bed for several multi-class sequence classification architectures. This gold-standard dataset was partitioned into a 90/10 train-validation split. Hyperparameter optimization was conducted via a Bayesian sweep for each model tested.

We benchmarked the traditional machine learning models SVM [17], Random Forest, Logistic Regression [18], XGBoost [19], and LightGBM [20]. We also benchmarked the transformer-based models, DistilBERT, ELECTRA [21], XLNet [26], and RoBERTa.

**Fig. 2:**
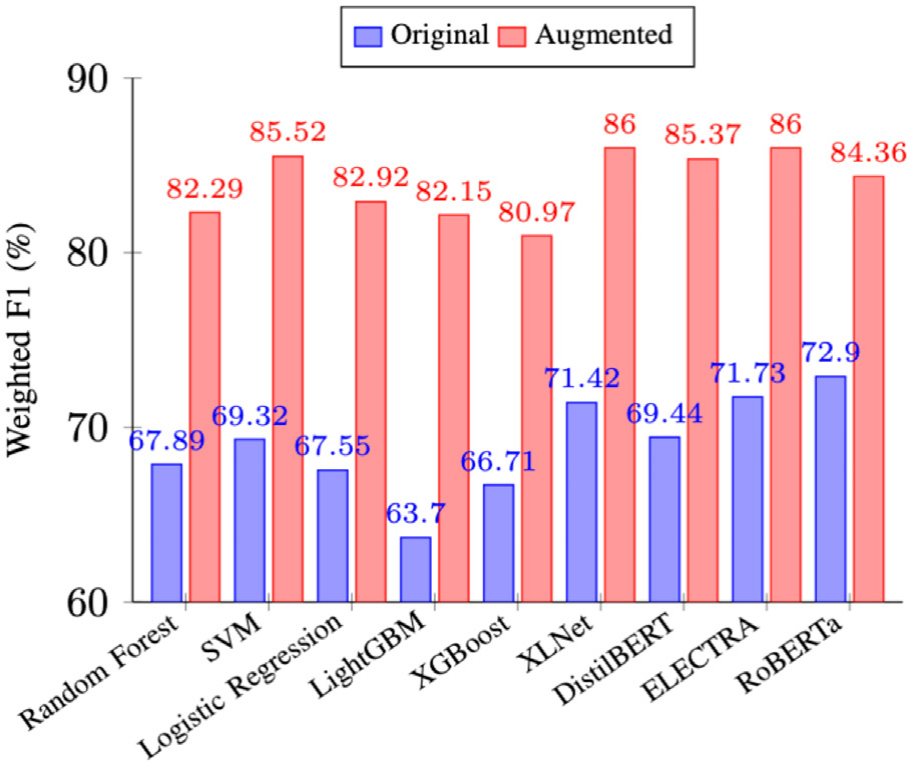
Model performance before and after back-translation augmentation.

Experimental results demonstrated that back translation significantly improved model performance across the board, (See Fig. 2). For example, the F1 score for SVM improved from 69.32% to 85.52% after being augmented by the back translation technique. XLNet was the overall best performing model with an 86.7% F1 score. Through testing we identified the optimal training configuration for XLNet; the AdamW optimizer, a learning rate of 3e-5, a training batch size of 8, and a duration of 10 epochs. Based on the results of these experiments, XLNet was the model selected to perform the weak supervision.

**Fig. 3:**
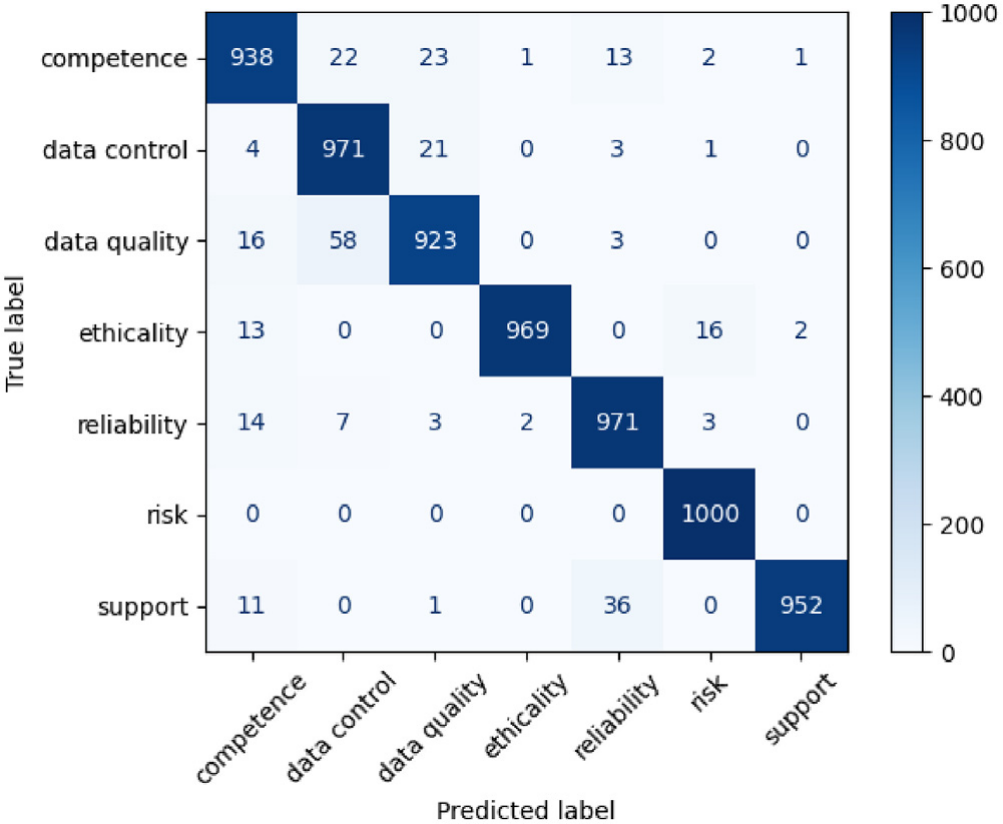
Confusion matrix of the seven classes on ground truth dataset with XLNet.

The confusion matrix (Fig. 3) illustrates the classification performance across the seven trust and privacy dimensions. The high concentration of values along the main diagonal indicates strong predictive accuracy for all classes, particularly for *Risk*, which achieved a perfect classification count of 1000.

### 4.5 Large-Scale corpus

An additional scrape was performed in April 2025 to expand the longitudinal scope and app coverage of the corpus. This second phase targeted 67 mHealth applications, (adding 8 new apps to the initial set), to ensure a comprehensive representation of the market. This effort resulted in a final consolidated corpus of 480,450 reviews spanning from 2011 to 2025.

### 4.6 Classification results

**Fig. 4:**
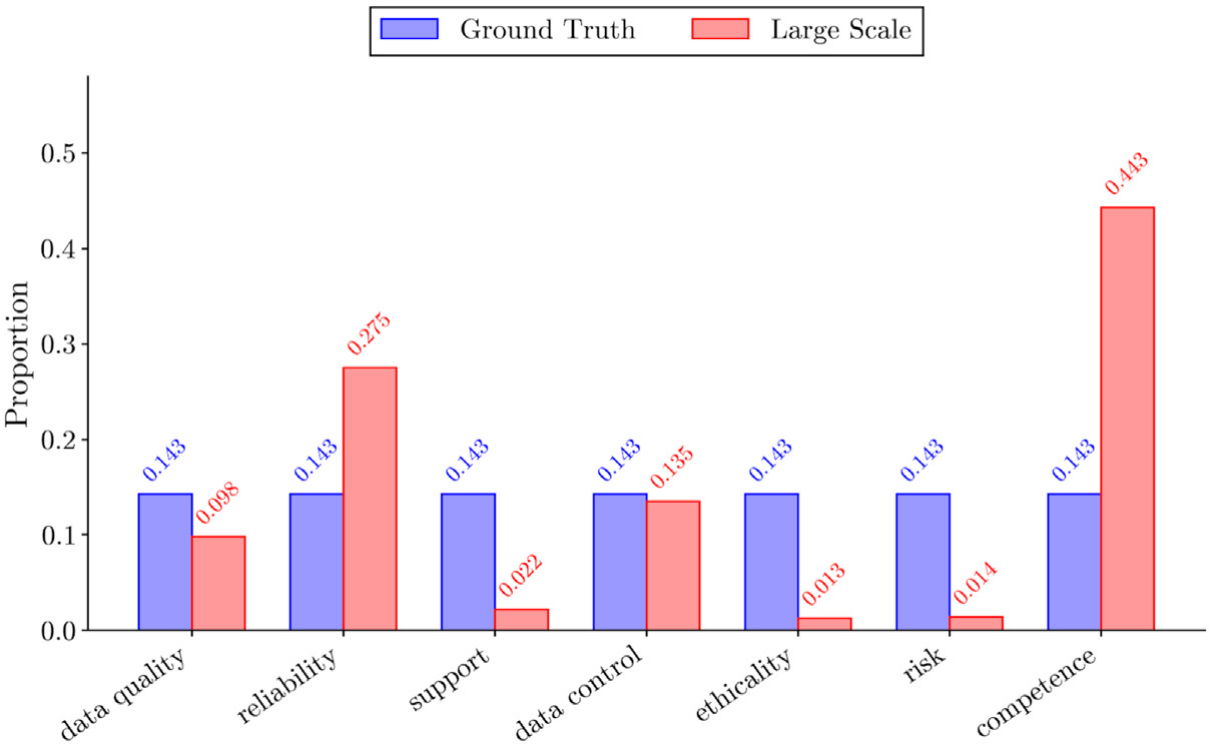
Class distribution of HARPT and ground truth dataset by proportion.

**Fig. 5:**
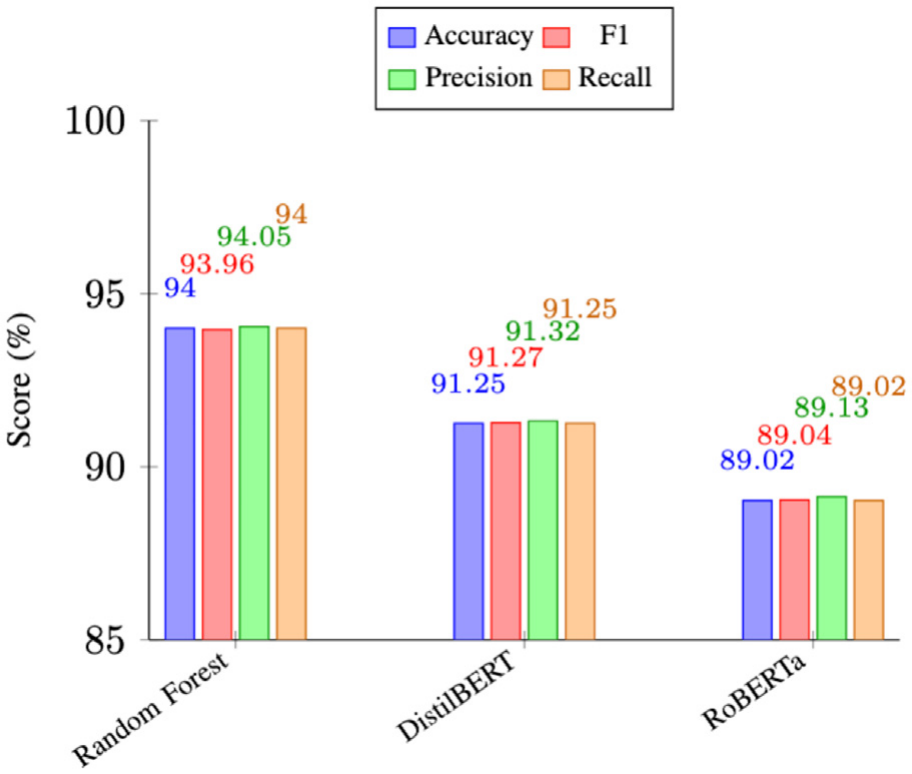
Performance comparison of models across evaluation metrics.

The fine-tuned XLNet model [22] was used to propagate the labels across the 480,450 unannotated reviews in the HARPT corpus using weak supervision. Inference was performed with a batch size of 64 and a maximum sequence length of 512 tokens. We applied softmax confidence scoring to the output logits to quantify the certainty of the model’s predictions. The quality of the labels in the HARPT corpus and performance of models on the dataset were evaluated. We benchmarked both traditional machine learning and transformer-based models, as shown in Fig. 5. The Random Forest [23] classifier had the highest overall performance with an accuracy of 94% and strong F1, precision and recall scores. The transformer-based models we evaluated, DistilBERT [24] and RoBERTa [25] also performed very well, with an accuracy of 91.25% and 89.02%, respectively. Fig. 4 shows the comparative class distributions between the ground truth and weakly labeled dataset. While ground truth is artificially balanced (∼14.3% per class) via back-translation, the large-scale corpus reflects true, real world user concerns. Real world distribution shows its heavily skewed toward clinical and technical performance, (Competence and Reliability dominating discourse), whereas abstract concerns such as Risk and Ethicality remain comparatively rare. Despite being a weakly labeled dataset, the consistently high performance across models suggests that the resulting labels are of excellent quality to support effective learning.

To evaluate the efficacy of our classification framework, all predictive results were explicitly linked to the performance of our chosen transformer architecture, XLNet. On the 10% gold-standard holdout validation split (700 reviews), the fine-tuned XLNet algorithm demonstrated exceptional predictive capability across all seven multidimensional classes, achieving an overall Macro F1 score of 0.96. The success of these classification results is directly attributable to XLNet's permutation-based autoregressive training. Unlike traditional masked language models, the XLNet algorithm excels at capturing complex, bidirectional context without relying on artificial mask tokens. This architectural advantage allowed the algorithm to accurately parse the highly unstructured language of mHealth users, successfully distinguishing nuanced, closely related categories with high mathematical confidence. Consequently, the high-fidelity pseudo-labels generated across the remaining 473,000 reviews during the weak supervision phase are directly rooted in the robust contextual embeddings produced specifically by the XLNet algorithm.

**Fig. 6:**
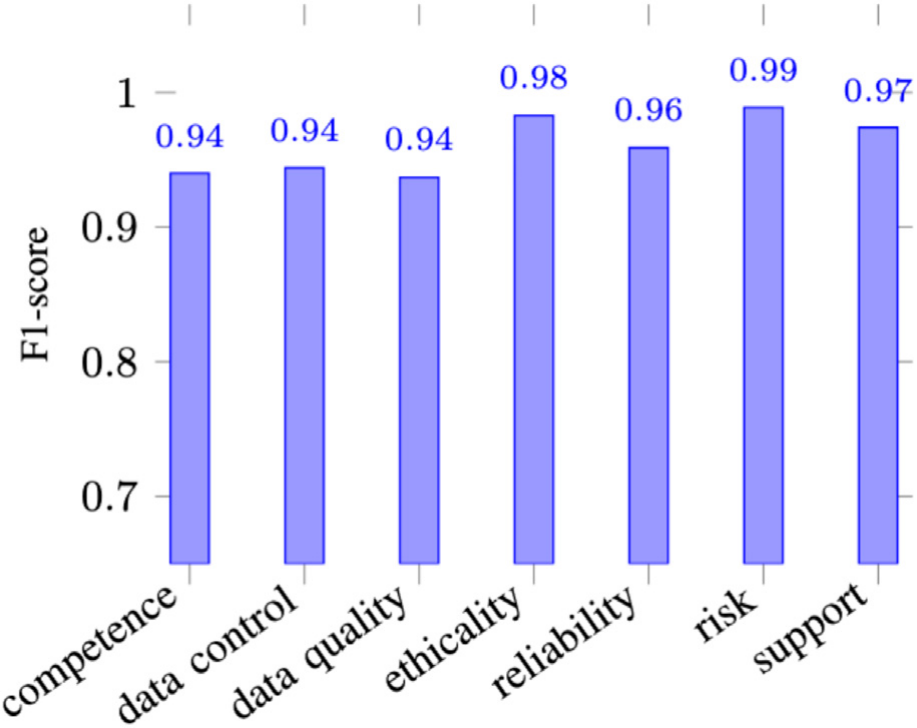
Per-class F1-scores on the ground truth dataset with XLNet.

This model also achieved high classification accuracy for minority classes (see Fig. 6), specifically *Risk* (= 0.99) and *Ethicality* (= 0.983). While *Competence* presented the highest relative complexity for the model, it still maintained a high score of 0.940.

Beyond standard performance metrics, the reliability of the weak supervision process was assessed through confidence calibration. As shown in Table 3, the model exhibits a high degree of calibration, where the predicted probability closely matches the empirical accuracy. Notably, in the highest confidence bin (0.9–1.0), which contains over half of the corpus predictions, the model achieves an accuracy of 98.3% with a negligible error of 0.3%.

**Table 3:** Confidence calibration analysis on HARPT dataset with XLNet.

Confidence Range	Samples	Accuracy	Avg. Confidence	Error
0.0–0.5	12	41.7%	46.1%	4.4%
0.5–0.6	27	66.7%	54.6%	12.0%
0.6–0.7	34	64.7%	65.5%	0.8%
.07–0.08	47	74.5%	74.7%	0.2%
.08–0.9	53	84.9%	86.7%	1.8%
0.9–1.0	827	98.3%	98.6%	0.3%

To understand how specific privacy and trust concerns correlate with overall user satisfaction, we analyzed the average app star rating across classes on both datasets.

**Fig. 7:**
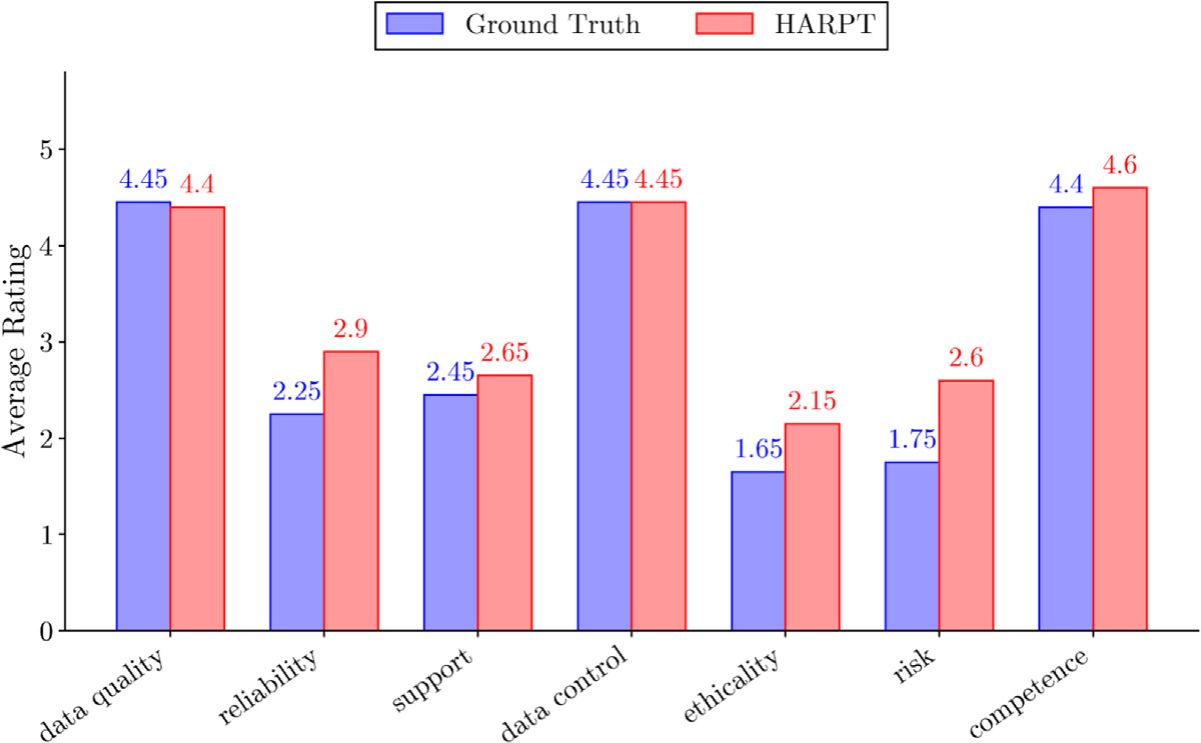
Star rating distribution of the 7 classes on HARPT and ground truth datasets.

The analysis revealed a distinct bifurcation in user satisfaction depending on the nature of the concern (See Fig. 7). Dimensions related to *Data Quality, Data Control*, and provider *Competence* were overwhelmingly associated with highly positive reviews, averaging approximately 4.5 out of 5 stars in both the ground truth and large-scale datasets. This suggests that when users discuss these specific dimensions, they are frequently affirming that the application or provider is performing these functions correctly.

Conversely, dimensions highlighting technical friction were strongly associated with negative user sentiment. In the ground truth dataset, reviews flagged for *Ethicality* and *Risk* exhibited the lowest average ratings, scoring approximately 1.6 and 1.8 respectively. Interestingly, when scaled across the remaining 473,000 reviews via weak supervision, the average ratings for these negative dimensions experienced a slight upward shift (*Risk* increased to approximately 2.6). This distribution shift indicates that while these four dimensions remain primary drivers of user dissatisfaction, the massive scale of the weakly supervised dataset captures a broader spectrum of moderate, nuanced feedback that is slightly less extreme but representative of the broader user population.

## 5 Applications and Use Cases

### 5.1 Research applications

The HARPT corpus and its classification framework have several high impact use cases for the research community. For NLP researchers, the 7000 gold standard dataset serves as a rigorous, domain-specific benchmark for finetuning and evaluating LLMs on user generated text. Besides model training, the large-scale HARPT corpus enables opinion mining and sentiment analysis specific to the digital health domain. The longitudinal nature of the data (spanning 14 years) allows behavioral and security researchers to conduct continuous trust and privacy monitoring, observing how user concerns evolve in response to macro-level data breaches or shifts in telehealth adoption. Finally, this taxonomy facilities data-driven requirement elicitation, allowing researchers to systematically map unstructured user reviews to concrete technical system requirements.

### 5.2 Industry and clinical applications

This framework also provides actionable, scalable tools for industry and clinical stakeholders. For mHealth app developers and UI/UX designers, the taxonomy acts as a diagnostic tool to pinpoint specific technical friction points, allowing them to remediate issues that actively degrade technology acceptance. For healthcare providers, the isolation of the Trust in Provider dimension offers a novel mechanism to monitor patient-provider relationships at scale. Health systems can identify instances where poor app infrastructure causes unintentional information blocking, thereby preventing technical failures from eroding clinical trust. For policymakers, this scalable NLP approach provides a mechanism to monitor compliance with data protection standards and identify emerging privacy threats within captive user populations that may require legislative intervention.

## Limitations

While the HARPT corpus provides a significant resource for mHealth research, users of the data should consider the following constraints.

The reviews were sourced exclusively from the Google Play Store. While this captures a massive user base, the dataset does not account for potential variations in user demographics or feedback styles present on other platforms, such as the Apple App Store.

Although the XLNet model achieved a Macro of 0.96 and high confidence calibration, the 480,450-review corpus is weakly labeled. Researchers requiring gold standard accuracy should utilize the 7000 ground-truth subset or filter the larger corpus by the provided confidence scores.

Despite implementing a rigorous three-pass rotating annotation workflow and achieving high inter-rater reliability, human annotation inherently introduces subjectivity. Annotators may unconsciously project their own baseline onto ambiguous textual feedback, meaning the gold standard labels remain approximations of the users original, unobservable intent.

The dataset is focused on English-language reviews. While back-translation used French, Spanish, and German for augmentation, the final corpus does not capture the cultural or linguistic nuances of non-English speaking mHealth users. While synthetic data was successfully generated via back-translation, it can occasionally introduce linguistic artifacts or subtly alter the semantic meaning of text, potentially smoothing over domain-specific nuances unique to user discourse.

Despite augmentation efforts for training, the real-world distribution of the 480k corpus remains heavily skewed toward *Competence* and *Reliability.* Users should apply appropriate sampling techniques if they intend to use the larger corpus for training new models.

Although our taxonomy is grounded in established frameworks, real world user feedback is rarely strictly compartmentalized. We observed inherent semantic overlap between closely related classes, such as Data Control and Data Quality. Because mHealth users frequently conflate technical bugs with institutional betrayal, this semantic blurring presents an ongoing challenge.

The propagation of labels across the remaining corpus via weak supervision relies on the assumption that XLNet model’s predictions accurately reflect ground truth. Pseudo-labeling inherently propagates a margin of machine-generated error at scale. Future research should explore human-in-the-loop active learning to continuously validate and refine these automated predictions.

## Ethics Statement

The authors have read and follow the ethical requirements for publication in *Data in Brief*. This work does not involve experiments with animals or direct intervention with human subjects. All data were collected from a public platform and have been fully anonymized to remove personally identifiable information, in compliance with the platform’s data redistribution policies.

## CRediT Author Statement

**Timoteo Kelly:** Conceptualization, Investigation, Methodology, Software, Writing – Reviewing & Editing, Writing – Original Draft, Data Curation. **Abdulkadir Korkmaz:** Data Curation, Writing – Reviewing & Editing, Software. **Samuel Mallet:** Data Curation, Software. **Connor Souders:** Data Curation, Software. **Sadra Aliakbarpour:** Data Curation. **Praveen Rao:** Supervision.

## Data Availability

DataverseHealth App Reviews for Privacy and Trust (HARPT) (Original data).

## Appendix Supplementary materials

Image, application 1

Image, application 2
