# Supplementary material for: Longitudinal datasets of health app reviews for privacy and trust modeling
Source: Data Brief. 2026 Apr 2;66:112740. doi: 10.1016/j.dib.2026.112740 (PMC13092470; doi:10.1016/j.dib.2026.112740)
Supplement: Supplementary file 1 [file mmc1.docx]

search_terms_data_quality = [

'Accurate', 'Precision', 'Correctness', 'Error-free', 'Valid', 'Reliable',

'Integrity', 'Trustworthy', 'Complete', 'Comprehensive', 'Inclusive',

'Exhaustive', 'Full', 'All-encompassing', 'Current', 'Updated', 'Recent',

'Timely', 'Modernized', 'Fresh', 'Data consistency', 'High-quality data',

'Well-maintained data', 'Clean data', 'Structured data', 'Standardized',

'Inaccurate', 'Outdated', 'Incomplete', 'Unreliable', 'Corrupted',

'Errors in data', 'Data issues', 'Data discrepancies', 'Data gaps',

'Missing data', 'Broken data', 'Mismatch', 'Misleading information',

'Stale data', 'Real-time updates', 'Lagging data', 'Delay in updates',

'Latency', 'Sensor reliability', 'Measurement errors', 'Tracking accuracy',

'Poor insights', 'Difficult to trust', "Results don't match",

'Untrustworthy data', 'Database errors', 'Backend issues', 'Sync problems',

'Corrupted files', 'Wrong information'

]

search_terms_data_control = [

'Accessibility', 'Retrieve', 'Obtain', 'View', 'Data access', 'User access',

'Permission', 'Edit', 'Modify', 'Correct', 'Update', 'Fix', 'Adjust',

'Amend', 'Revise', 'Delete', 'Remove', 'Erase', 'Expunge', 'Eliminate',

'Purge', 'Stop', 'Halt', 'Cease', 'Terminate', 'End', 'Pause', 'Control',

'Ownership', 'Manage', 'Authority', 'Rights', 'Autonomy',

'Restricted access', 'Limited control', 'Data unavailable', 'Locked data',

'Unable to edit', 'Cannot delete', 'Grant access', 'Revoke access',

'Access denied', 'Permission denied', 'View permissions', 'Edit rights',

'User autonomy', 'Data management', 'Change permissions', 'Control data',

'Data portability', 'Export data', 'Download data', 'Access my data',

'Right to correct', 'Right to delete', 'Opt-out', 'Opt-in', 'Data withdrawal',

'Manage permissions', 'Ownership rights', 'Control over data', 'User empowerment',

'Revocation of rights', 'Request deletion', 'Privacy settings',

'Data deletion request', 'Adjust access', 'Self-service data control',

'Custom permissions', 'Data locked', 'Cannot retrieve', 'Restricted editing',

'Request updates', 'Limited access', 'Editable fields', 'Non-editable data'

]

search_terms_ethicality = [

'Ethical', 'Honest', 'Transparent', 'Fair', 'Trustworthy', 'Accountable',

'Respectful', 'Responsible', 'Principled', 'Integrity', 'Upright', 'Moral',

'Conscientious', 'Reliable', 'Fair treatment', 'Confidential', 'Privacy protection',

'User consent', 'Data security', 'Transparency in data use', 'Clear terms of service',

'Respect for privacy', 'User rights', 'Fair policies', 'Non-exploitative',

'Non-manipulative', 'Open communication', 'Genuine', 'Authentic', 'Fair pricing',

'Honest marketing', 'Inclusive practices', 'Equal treatment', 'Equitable service',

'Clear consent', 'Responsible use of data', 'Non-discriminatory', 'Respectful service',

'Ethical standards', 'Compliance with regulations', 'Honest feedback', 'User protection',

'Non-deceptive', 'Good intentions', 'User advocacy', 'Fair practices', 'Ethical provider',

'High moral standards', 'Values-driven', 'Ethical approach', 'User-focused', 'Accountable provider',

'Ethics', 'Morality', 'Fairness', 'Accountability', 'Adherence', 'Compliance',

'Code of conduct', 'Guidelines', 'Standards', 'Proper practices', 'Professionalism',

'Best practices', 'Reliable behavior', 'HIPAA', 'HIPPA', 'Trustworthy interactions',

'Unethical', 'Dishonest', 'Misleading', 'Deceptive', 'Exploitative', 'Manipulative',

'Unfair', 'Intransparent', 'Hidden terms', 'Breach of privacy', 'Privacy violation',

'Unreliable information', 'Data misuse', 'Violating user rights', 'Unauthorized data collection',

'Non-compliant', 'Discriminatory', 'Irresponsible', 'Unaccountable', 'Fraudulent',

'Exploiting users', 'Misrepresentation', 'False advertising', 'Unclear terms',

'Unjust policies', 'Unfair treatment', 'Lack of transparency', 'Data leakage',

'Consent violations', 'Non-disclosure', 'Misleading information', 'Lack of user protection',

'Privacy breaches', 'Abusive practices', 'Unlawful behavior', 'Immoral', 'Corruption',

'Ethical principles', 'Moral integrity', 'Ethical dilemmas', 'Breach of ethics',

'Moral behavior', 'Unjust practices', 'Moral conduct', 'Ethical guidelines',

'Code violations', 'Improper behavior', 'Respectful practices', 'Social responsibility',

'Exploitation of users', 'Deceptive practices', 'Breach of trust', 'Abuse of data',

'Unjust treatment', 'Equality', 'Bias', 'Non-discrimination', 'Honest communication',

'Abuse of power', 'Predatory practices'

]

search_terms_competence = [

'Effective', 'Efficient', 'Reliable', 'Capable', 'Skilled', 'Experienced',

'Knowledgeable', 'Professional', 'Competent', 'Proficient', 'Expert',

'Technologically advanced', 'Innovative', 'State-of-the-art', 'Cutting-edge',

'Expertise', 'Mastery', 'Trustworthy provider', 'Solution-oriented',

'Problem-solving', 'Quality technology', 'High-performance', 'Dependable',

'Advanced solutions', 'Reliable technology', 'Well-designed', 'Effective solution',

'Capability', 'Successful implementation', 'Well-engineered', 'Well-built',

'User-friendly', 'Smooth operation', 'Streamlined', 'Intuitive', 'Proven track record',

'Established provider', 'Skilled team', 'Top-tier service', 'Industry-leading',

'Reputable', 'Strong track record', 'Satisfactory performance', 'Dependable service',

'Efficient technology', 'High-quality solutions', 'Sustained performance',

'Friendly service', 'Caring provider', 'Supportive', 'Helpful', 'Patient',

'Attentive', 'Considerate', 'Understanding', 'Compassionate', 'Approachable',

'Empathetic', 'Personalized care', 'Responsive', 'Easy to communicate with',

'Clear communication', 'Comfortable experience', 'Positive interaction',

'Welcoming environment', 'Accessible support', 'Prompt responses', 'Respectful',

'Thorough explanations', 'Engaged provider', 'Friendly staff', 'Kind service',

'Unhelpful', 'Unfriendly', 'Apathetic', 'Difficult to communicate with',

'Unresponsive', 'Inconsiderate', 'Impersonal', 'Dismissive', 'Rushed service',

'Lack of support', 'Lack of care', 'Cold interaction', 'Insensitive',

'Unapproachable', 'Confusing instructions', 'Poor communication', 'Unclear explanations',

'Frustrating experience', 'Lack of empathy', 'Unprofessional behavior',

'Unreliable service', 'Ineffective support', 'Low-quality care', 'Indifferent service'

]

search_terms_reliability = [

'Reliable', 'Consistent', 'Stable', 'Dependable', 'Steady', 'Trustworthy',

'Resilient', 'Solid performance', 'High uptime', 'Available', 'Durable',

'Robust', 'Persistent', 'Uninterrupted', 'Continuous', 'Well-maintained',

'Dependable technology', 'Sustained performance', 'Long-term reliability',

'Proven reliability', 'Reliable service', 'Performance consistency', 'High availability',

'No downtime', 'Fast recovery', 'Error-free operation', 'Smooth operation',

'Performance stability', 'Fast response time', 'Quick resolution', 'On-demand availability',

'Sustained availability', 'Responsive', 'Always accessible', 'Seamless experience',

'Reliable connectivity', 'Constant performance', 'Stable service', 'Unfailing',

'Always functioning', 'Dependable system', 'Efficient system', 'Consistent uptime',

'Flawless service', 'Optimized performance',

'Unreliable', 'Inconsistent', 'Unstable', 'Failure', 'Outage', 'Downtime',

'Crashes', 'Glitches', 'Intermittent', 'Lagging', 'Delays', 'Performance issues',

'Disruptions', 'Slow response', 'Inaccessible', 'Unresponsive', 'Service interruptions',

'Faulty', 'Broken', 'Malfunctioning', 'Unpredictable', 'Bugs', 'Unreliable service',

'Data loss', 'Inavailability', 'Connection errors', 'Technical problems',

'Issues with uptime', 'Slow performance', 'Inconsistent access', 'Unstable connection',

'Frequent crashes', 'Inconsistent service'

]

search_terms_support = [

'Support', 'Customer service', 'Help', 'Assistance', 'Technical support', 'Troubleshooting',

'Guidance', 'Resources', 'FAQ', 'User manual', 'Knowledge base', 'Live chat',

'Help desk', 'Support team', 'Customer care', 'Onboarding', 'Tutorial', 'User guides',

'Documentation', 'Self-service', 'Support portal', 'Help center', 'Support resources',

'Accessibility', 'Responsive support', '24/7 support', 'Multilingual support', 'Instant support',

'Easy access to support', 'Prompt support', 'Effective support', 'Friendly support',

'User-friendly help', 'Clear instructions', 'Supportive staff', 'Personalized support',

'Support tickets', 'Community support', 'Remote assistance', 'Comprehensive support',

'Guided assistance', 'Quick resolution', 'Proactive support', 'Expert assistance',

'Immediate help', 'Technical assistance', 'Easy-to-reach support', 'Available support',

'On-demand support', 'Customer-focused support', 'Efficient troubleshooting', 'Timely assistance',

'Problem-solving support', 'Well-trained staff', 'Support availability', 'Assistance channels',

'In-app support', 'Phone support', 'Email support', 'Chat support', 'Support response time',

'Unresponsive support', 'Lack of support', 'Unhelpful support', 'Slow response',

'Inadequate support', 'Poor customer service', 'Limited support', 'No support',

'Hard to reach support', 'Unfriendly support', 'Unclear instructions', 'Unresolved issues',

'Lack of guidance', 'Limited resources', 'No troubleshooting', 'Unhelpful documentation',

'Technical issues unresolved', 'No follow-up', 'Ignored support tickets', 'Lack of communication',

'Support delays', 'Poor resolution', 'Confusing help', 'Difficult to access support',

'No clear help available', 'Unprofessional support', 'No response from support', 'Frustrating support',

'Unreliable support', 'Ineffective troubleshooting', 'Unresolved complaints',

'Limited contact options', 'Unskilled staff', 'Unresolved technical issues'

]

search_terms_risk = [

'Risk', 'Concern', 'Fear', 'Uncertainty', 'Danger', 'Potential harm', 'Privacy risk',

'Data security risk', 'Security breach', 'Health risk', 'Financial risk', 'Trust risk',

'Risk of misuse', 'Data leakage', 'Vulnerability', 'Threat', 'Cybersecurity risk',

'Unpredictable outcomes', 'Risk of fraud', 'Exposure to scams', 'Loss of privacy',

'Risk of exploitation', 'Risk of harm', 'Data breach', 'Data theft', 'Unauthorized access',

'Malware risk', 'Phishing', 'User exploitation', 'Exploitation', 'Loss of control', 'Addiction risk',

'Unclear terms', 'Terms of service risk', 'Misuse of data', 'Unintended consequences',

'Negative impact', 'Negative side effects', 'Invasion of privacy', 'Unanticipated risks',

'False promises', 'Fake claims', 'Risky technology', 'Unwanted outcomes', 'Potential misuse',

'Dangerous practices', 'Risk of error', 'Operational risks', 'Unethical risk',

'System failures', 'Unreliable outcomes', 'Privacy concerns', 'Lack of control',

'Unsecure platform', 'Doubtful technology', 'Risk of addiction', 'Technology dependency',

'Overreliance on technology', 'Reputation risk', 'Legal risks', 'Unethical risks',

'Ethical concerns', 'Lack of transparency', 'Liability risk', 'Risk of non-compliance',

'Financial loss', 'Unpredictable consequences', 'Risk of failure', 'Risks of misuse'

]
