# Supplementary material for: Longitudinal datasets of health app reviews for privacy and trust modeling
Source: Data Brief. 2026 Apr 2;66:112740. doi: 10.1016/j.dib.2026.112740 (PMC13092470; doi:10.1016/j.dib.2026.112740)
Supplement: Supplementary file 2 [file mmc2.docx]

<https://huggingface.co/tk648/XLNet-base-finetuned-HARPT>
